# Dynamic changes of otolith organ function before and after repositioning in patients with benign paroxysmal positional vertigo detected by virtual reality auxiliary technology: A cohort study

**DOI:** 10.3389/fneur.2022.1007992

**Published:** 2022-11-08

**Authors:** Chunjie Zhao, Qingjun Yang, Jijun Song

**Affiliations:** Department of Otolaryngology, Head and Neck Surgery, Zhou Kou Central Hospital, Zhoukou City, China

**Keywords:** BPPV, otolith organ function, virtual reality auxiliary, subjective visual vertical, subjective visual horizontal, dynamic unilateral centrifugation, dynamic change

## Abstract

**Objectives:**

To dynamically investigate otolith function in patients with benign paroxysmal positional vertigo (BPPV) before, after, and 1 month after repositioning, and explore the possible compensation mechanisms.

**Methods:**

Thirty-six patients confirmed with BPPV (canal lithiasis) treated in our hospital between August 2020 and March 2021, as well as 36 health controls matched for age and gender (normal control group, NC group) were enrolled. For NC group, the virtual reality (VR) auxiliary static subjective visual vertical (SVV), subjective visual horizontal (SVH), and SVV of dynamic unilateral centrifugation (DUC), were measured at inclusion. For the BPPV group, visual analog scale (VAS) was used to assess the vertigo degree, while static SVV, SVH, and DUC were performed before, after, and 1 month after repositioning. First, we compare the deviations of SVV0/SVH0° when the subject's head is in the positive position, and SVV of DUC between BPPV and NC groups before repositioning, after which we compared the deviations in SVV45, SVV90, SVH45, SVH90°, and SVV of DUC between the affected and unaffected sides before repositioning. Finally, paired *t*-test was used to compare the VAS score, deviations in static SVV0, SVV45, SVV90, SVH0, SVH45, and SVH90°, and deviations in SVV of DUC before, after, and 1 month after repositioning. (Here, 0, 45, and 90° refer to the angle which the center axis of head deviates from the gravity line.)

**Results:**

SVV0 SVH0°, and SVV of DUC at 120 and 180°/s 0 significantly differed between BPPV and NC group before repositioning. The deviations in SVV45, SVV90, SVH45, SVH90°, and SVV of DUC at 120°/s-2 and 180°/s-4.5 did not significantly differ between bilateral sides in BPPV patients before repositioning. The deviation in SVH90° was significantly lower after repositioning than before. The deviation in SVH45° was significantly higher 1 month after repositioning than before. The deviation angle of SVV of DUC at 180°/s-0 was significantly lower after repositioning than before. The vertigo VAS score of patient with BPPV continued to decrease after repositioning.

**Conclusion:**

Before repositioning, the otolithic organ function of BPPV patients was obviously impaired, with no significant difference between the healthy and affected ear. After repositioning, there was a transient recovery of otolithic organ dysfunction followed by a sustained decline to similar levels to before repositioning.

## Introduction

Benign paroxysmal positional vertigo (BPPV) is a peripheral vestibular disorder with repeated transient vertigo and typical nystagmus that is induced by changes in head position relative to the direction of gravidity. BPPV accounts for 20–30% of vestibular vertigos. It has a very high prevalence in individuals who are 40–59 years old, while the male-to-female rate is approximately 1:1.5–1:2.0 (1). The pathogenesis of BPPV remains unclear; however, the most acknowledged theories include canalolithiasis and cupulolithiasis (2), while the dislodgment of otolith particles from otolith organ (*utricular maculae*), the peripheral vestibular receptor, has been considered as the major pathogenesis. Specific particle repositioning maneuvers could rapidly alleviate nystagmus and vertigo in most patients (3–5). Yet, studies have shown that the recurrence rate of BPPV is as high as 37–67.3%. In addition, the recurrence could influence both the affected and unaffected side, while approximately 56% of the recurrences occur within 1 year after repositioning (6, 7). Only very few studies have investigated the functions of otolith organ after repositioning in BPPV patients.

Utriculus (otolith organ) mainly senses linear acceleration and gravity (8) and is the potential pathogenic site of BPPV. Currently, the methods that indirectly assess the functions of utriculus include vestibular evoked myogenic potentials (VEMPs), subjective visual vertical (SVV), and subjective visual horizontal (SVH). SVV and SVH refer to the perception of humans to gravitational vertical and gravitational horizontal lines in a dark environment, respectively, which serves to rule out the influences of visual references. SVV and SVH are rapid and convenient methods for assessing the functions of utriculus, and the preset angle could reflect the asymmetry and balancing of static tensions of bilateral utriculus (9–12). Currently, a simple device called “bucket test” is generally used for the examination at the midline of the head of 0° in the static state and the dynamic examinations during off-axis rotations (13). Yet, few studies reported using virtual reality (VR) glasses to assist the examination of dynamic and static SVV and SVH. In addition, some scholars have found that dynamic SVV examination is more sensitive than static examination (14, 15), that is, SVV is examined simultaneously with centrifugal acceleration stimulation of otolith, namely dynamic unilateral centrifugation (DUC). The vestibular otolith organs are symmetrical organs located in the temporal bones on both sides of the cranial sagittal plane. When the center of the head is 3.87 cm away from the rotation axis to the left or right, the utricle on one side is located at the rotation axis, and the other side is deviated from the rotation axis by 7.74 cm. When the distance between the center of the head and the axis of rotation exceeds this distance, the utricles on both sides will be stimulated, but the stimulation amount is different. Off-axis rotation SVV can be detected when the person deviates from the axis and rotates around the axis, also known as off-axis rotation, or unilateral centrifugal force detection. During the detection, the tested ear is 7–8 cm away from the axis, and the contralateral ear is located at the axis and rotates at a constant speed at a certain speed. The vestibular eye movement response of the horizontal semicircular canal disappeared during constant rotation, centrifugal force was generated by off-axis rotation, or linear acceleration stimulated the utricle located off-axis. Unlike the rotation around the axis (bilateral centrifugal force), only the detection ear (located off the axis) is affected by the gravitational inertia force (GIF) formed by the unilateral centrifugal force. People with normal vestibular function are affected by unilateral centrifugal force, and the SVV skew values on both sides are symmetrical. When left ear is off-axis, namely left utricle is stimulated, SVV tilts to the right. When right ear is off-axis, namely right utricle is stimulated, SVV is left tilt (36).

A series of studies have also been conducted on DUC before. Gonzalez Set the translation time of UC-SVV rotation axis as 5, 10, 15, At 20, 25, and 30 s, 43 young healthy volunteers were randomly divided into groups for peak The UC-SVV test at a speed of 300° / s found that the rotation axis Short translation time will lead to greater variation in test results (16). Clarke, etc. It is considered that the GIA can be operated only when the rotation peak speed reaches 300 −400° / s. The unilateral elliptic sac produces marked excitation. The rotation axis peaks at 300° / s Speed translation of 3.5 cm centrifugal rotation will yield 0.192 g (1.9 m/s) the angular deflection can reach 11.3° (17). Chen Taisheng et al. performed UC-SVV with a rotation speed of 60°/s and a displacement of 3.85 cm in the normal population. After repeated examination, the average value was taken, and the results were in line with normal distribution (18).

Therefore, this study compared the SVV and SVH at different preset angles of the head, as well as the changes in SVV of dynamic unilateral centrifugation (DUC in patients with unilateral idiopathic BPPV). We also explored the dynamic changes in the function of the otolith organ before and after repositioning in canal BPPV patients. Our findings provide theoretical evidence for clinical diagnosis and treatment of BPPV, as well as investigations of possible pathogenic and compensatory mechanisms of BPPV.

## Subjects and methods

### Subjects

A total of 36 patients with unilateral idiopathic canal BPPV who were treated in the Otolaryngology Department of Zhoukou Central Hospital between August 2020 and March 2021 were included in this study. The inclusion criteria were as follows (19): (1) with the chief complaint of head position change induced transient vertigo, lasting < 1 min; (2) Dix-Hallpike test or roll test could induce vertigo and typical nystagmus, characterized by latent, transient, fatigability, and convertibility; (3) the type of nystagmus was in agreement with the presentations of affected semicircular canal; and (4) informed consents were obtained from the patients or the families. The exclusion criteria were: (1) with a previous history of vestibular diseases or ear diseases; (2) with a history of any type of dizziness or vertigo before; (3) cranial MRI examination showed disorders of the central nervous system (CNS); (4) could not cooperate during the examinations or participate in follow up due to other severe diseases or cognition impairment.

Thirty-six concurrent healthy adults matched for age and gender were enrolled as the controls (NC group). The inclusion were: (1) with no history of dizziness or vertigo, equilibrium disorder, hearing disorder, or otitis media; with no nervous system or skeletal system diseases; with no closure or open cranial trauma history; (2) with good neck movement ability; (3) with normal visual acuity or the corrected visual acuity of ≥1.0; (4) could understand and cooperate in the examinations, and those who signed informed consents. The exclusion criteria were as follows: (1) with a history of dizziness or vertigo; (2) with hearing disorders; (3) with eye diseases; (4) could not understand the study or could not cooperate in the study.

### Epidemiology, clinical characteristics, and VAS score of vertigo

Clinical data including age, gender, side of the disease, site of disease, time from disease onset to diagnosis, pure tone audiometry (PTA) results; accompaniment of chronic diseases (such as hypertension, diabetes, and hyperlipidemia) before the treatment were collected for all the subjects. Visual Analog Scale (VAS) was used to assess the severity of vertigo symptoms (0–10), which was performed for the included BPPV patients before, after, and 1 month after repositioning.

### SVV, SVH, and dynamic unilateral centrifugation

Static SVV and SVH, as well as DUC were performed before, after, and 1 month after repositioning for all BPPV patients, while for healthy controls, they were performed only at the time of inclusion.

Epley maneuver was performed for patients with posterior semicircular canal BPPV, and the Barbecue maneuver was performed for patients with horizontal semicircular canal BPPV. Dix-Hallpike test was performed again for all the patients 30 min after repositioning, and the disappearance of nystagmus and vertigo indicated the success of the repositioning maneuver.

VR auxiliary technology was used for the examination of static SVV and SVH, as well as SVV of DUC at 120 and 180°/s for all the subjects. All the tests were performed by experienced laboratory physicians engaged in clinical practices for at least 5 years. For the examinations of SVV and SVH, the subjects were asked to wear VR glasses (Yougeng ZT-VNG-I), after which the examinations were performed at the following fixed angles of head: midline position (0°), left tilted for 45°, left tilted for 90°, right tilted for 45°, and right tilted for 90°. Two pre-examinations were firstly performed to familiarize patients with the examination procedures, after which five measurements were acquired at each head position, and the mean values were calculated. For the one-sided centrifugation experiment, the subjects were asked to sit in a high-frequency, high-speed rotating chair (Yougeng ZT-VNG-I). The rotation was started from the central vertical axis, and the speed was gradually increased to 120 or 180°/s and then maintained at a constant speed. The rotation was stopped when the stimulation of bilateral semicircular canals appeared, and the SVV deviation was recorded. Afterward, rotations at the constant speeds of 120 and 180°/s were performed, and the present angles of left/right SVV at different rotation speeds were measured when the vertical axis horizontally deviated to left/right for 3.9 cm from the central.

### Ethics statement

The Zhou Kou Central Hospital Ethical permission committee approved study (AF-HEC-006-02.0) and all subjects provided their informed consents.

### Statistical analysis

SPSS25.0 software (IBM, Armonk, NY, USA) was used for all statistical analyses. Consecutive data were described by means and standard deviations. The *t*-test was used for the comparison of consecutive data between different groups, and the chi-square test was used for the comparison of qualitative data between different groups. The dynamic changes in static SVV and SVH at different head positions, SVV of DUC (120 and 180°/s), and dynamic changes of VAS scores of vertigo in BPPV patients were analyzed by paired *t*-test. *P* < 0.05 was considered statistically significant.

## Results

### Epidemiological and clinical characteristics

A total of 36 BPPV patients, 13 males and 23 females, with a mean age of 46.75 ± 10.327 years (23–63 years), were included in this study. The time from disease onset to diagnosis of these patients was 8.94 ± 8.672 d (2–30 d). Among them, 10 were with the disease on the left side and 26 on the right side, respectively. Eight patients were with the disease in the horizontal semicircular canal, and 28 patients were with the disease in the posterior semicircular canal. The effect of repositioning was good in 32 patients and suboptimal in four patients (Table 1).

**Table 1:** Epidemiological and clinical data of the 36 BPPV patients.

	**x ±σ/percentage**
Age (year)	46.75 ± 10.327
Sex (female)	23/36
Lateral (left)	10/36
Onset to therapy	8.94 ± 8.672
Horizontal semicircular canal	8/36
Posterior semicircular canal	28/36
Effect of otolith reduction treatment (well)	32/36

### Comparison of otolith organ functions in BPPV patients before and after repositioning

An Independent *t*-test was used to analyze whether the deviations of SVH0, SVV0°, and SVV of DUC at 120 and 180°/s 0 were significantly different between the BPPV group and the age and gender-matched NC group. We found that compared with the NC group, the deviations in SVH0, SVV0°, and SVV of DUC at 120 and 180°/s 0 were significantly higher in the BPPV group, indicating the presence of otolith organ dysfunction in the BPPV group (Table 2).

**Table 2:** Comparison of deviations in SVH, SVV, and DUC-120°/s 0 and 180°/s 0 SVV between the BPPV and NC group before repositioning.

	**BPPV (*n* = 36)**	**NC (*n* = 36)**	***p***
SVH0° (°) x ±σ	1.854 ± 1.815	0.993 ± 0.792	0.012*
SVV0° (°) x ±σ	2.709 ± 1.979	1.541 ± 1.661	0.008*
DUC at 120°/s 0	2.527 ± 2.099	1.677 ± 0.960	0.032*
DUC at 180°/s 0	3.622 ± 2.676	1.677 ± 1.656	0.000*
Sex (female)	23/36 (63.89%)	22/36 (61.11%)	0.808
Age (year)	46.75 ± 10.327	47.33 ± 10.312	0.811

* *p* < 0.05.

### Comparison of otolith organ functions between the affected and unaffected sides

An independent *t*-test was used to investigate whether the deviations in SVV45°, SVV90, SVH45°, SVH90°, and SVV of DUC at 120°/s-2 and 120°/s-4.5 in BPPV patients were significantly different between the affected and unaffected sides before repositioning. The findings showed that the deviations in S VV45, SVV90, SVH45°, SVH90°, and SVV of DUC at 120°/s-2 and 120°/s-4.5 did not significantly differ between the two sides in BPPV patients, indicating that the otolith organ functions were not significantly different between the affected and unaffected sides in BPPV patients (Table 3).

**Table 3:** Comparison of deviations of SVV45°, SVV90°, SVH45°, SVH90°, and DUC-120°/s-2, DUC-180°/s-4.5 SVV between the affected and unaffected sides in BPPV patients before repositioning.

	**Affected side**	**Healthy side**	***p***
SVV45° (°)	5.171 ± 4.1408	6.006 ± 4.800	0.432
SVV90 (°)	6.972 ± 7.064	6.947 ± 5.204	0.978
SVH45° (°)	5.661 ± 4.911	7.213 ± 5.600	0.215
SVH90° (°)	6.456 ± 6.097	5.306 ± 5.722	0.412
DUC at 120°/s-2	3.163 ± 2.484	2.982 ± 2.588	0.763
DUC at 180°/s-4.5	3.788 ± 3.468	4.182 ± 2.718	0.593

### Dynamic changes of otolith organ functions before and after repositioning in BPPV patients

**Figure 1:**
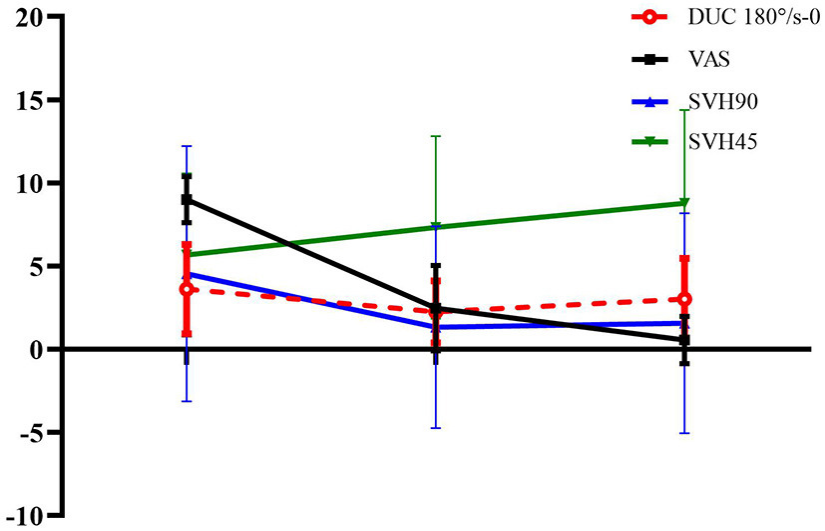
Dynamic changes of otolith organ functions in BPPV patients.

Paired *t*-test was used to compare the deviations in SVV0°, SVH0°, and SVV of DUC 120°/s-0 and 180°/s-0 of BPPV patients, as well as deviations in SVV45°, SVV90, SVH45°, SVH90°, SVV of DUC at 120°/s-2 and 180°/s-4.5, and VAS score of the affected side among different time points, i.e., before, after, and 1 month after repositioning. The findings showed that compared with before repositioning, the deviation in SVH90° and SVV of DUC at 180°/s-2 were significantly lower immediately after repositioning, and the VAS score was also significantly reduced. Compared with before repositioning, the deviation in SVH45° significantly increased, while VAS score significantly decreased 1 month after repositioning (Figure 1). These findings indicated that the otolith organ functions could be transiently restored immediately after repositioning in BPPV patients, and the vertigo degree also decreased continuously thereafter, while the otolith organ functions continued to reduce until reaching similar levels as those before repositioning (Table 4).

**Table 4:** Changes of deviations of SVV0°, SVV45°, SVV90°, SVH0°, SVH45°, SVH90°, and DUC-120°/s-0/2 and 180°/s-0/4.5 SVV before, after, and 1 month after repositioning.

	**Before x ±σ**	**After x ±σ**	**p**	**Before x ±σ**	**1 month later x ±σ**	***p***
SVH0°	1.854 ± 1.815	1.657 ± 1.159	0.593	1.854 ± 1.815	2.288 ± 2.297	0.275
SVH45°	5.661 ± 4.911	7.330 ± 5.487	0.111	5.661 ± 4.911	8.767 ± 5.609	0.006*
SVH90°	6.456 ± 6.097	3.961 ± 4.749	0.029*	6.456 ± 6.097	4.770 ± 4.643	0.102
SVV0°	2.709 ± 1.979	2.341 ± 1.910	0.312	2.709 ± 1.979	2.666 ± 1.658	0.904
SVV45°	5.171±± 4.141	6.991 ± 6.593	0.144	5.171 ± 4.141	5.855 ± 5.171	0.495
SVV90	6.972 ± 7.064	7.685 ± 6.586	0.485	6.972 ± 7.064	7.708 ± 6.706	0.536
120°/s-0	2.527 ± 2.099	2.239 ± 1.751	0.481	2.527 ± 2.099	2.116 ± 2.989	0.522
120°/s-2	3.163 ± 2.484	2.605 ± 2.036	0.236	2.527 ± 2.099	3.385 ± 2.762	0.123
180°/s-0	3.622 ± 2.676	2.251 ± 1.860	0.008*	3.622 ± 2.676	3.024 ± 2.453	0.321
180°/s-4.5	3.788 ± 3.468	3.072 ± 2.435	0.164	3.788 ± 3.468	3.543 ± 2.525	0.714
VAS	9.00 ± 1.394	2.47 ± 2.569	0.000	9.00 ± 1.394	0.56 ± 1.423	0.000

* *p* < 0.05.

## Discussion

In this study, the changes in SVV, SVH, and SVV of DUC at different head positions were compared between patients with unilateral idiopathic canal BPPV and healthy controls. The findings showed that the SVV0, SVH0°, and SVV of DUC at 120°/s 0 and 180°/s 0 significantly differed between BPPV patients and healthy controls before repositioning. The deviations in SVV45°, SVV90, SVH45°, SVH90°, and DUC at 120°/s-2 and 180°/s-4.5 were not significantly different between the two sides in BPPV patients before repositioning. The deviation in SVH90° was significantly lower after repositioning compared with the preset angle before repositioning. The deviation in SVH45° was significantly higher 1 month after repositioning compared with the preset angle before repositioning. The deviation in SVV of one-sided centrifugation experiments at 180°/s 0 was significantly lower after repositioning than before repositioning. However, the deviation in SVV of DUCC at 180°/s 0 increased; yet, statistical significance was not reached at 1 month after repositioning, compared with immediately after repositioning, and was not significantly lower than before repositioning.

### Otolith organ dysfunction was present in BPPV patients before repositioning

BPPV is the most common cause of peripheral vestibular vertigo (1, 19). The dislodgment of otoconia from the utriculus into a semicircular canal in BPPV patients is considered the most common cause of semicircular canal-canal BPPV (20). Various autopsy studies have shown that patients with BPPV are generally accompanied by injuries of utricular maculae (21). The findings of this study showed that the deviations in SVV0°, SVH0°, and SVV of DUC (120°/s-0/2 and 180°/s-0/4.5) before repositioning significantly differed between BPPV patients and healthy controls matched for age and gender, indicating the presence of otolith organ dysfunction in BPPV patients. Consistent with our findings, a study performed by Munetaka Ushio et al. in 2007 revealed that canal-BPPV patients were accompanied by otolith organ dysfunction, and ~82.1% (23/82) p-BPPV (posterior semicircular canal-BPPV) patients were with the deviation of SVH toward the unaffected side (22).

### The otolith organ functions were not significantly different between the bilateral sides in BPPV patients

The results of this study showed that the deviations of SVV45°, SVV90, SVH45°, SVH90°, and SVV of DUC at 120°/s-2 and 180°/s-4.5 did not significantly differ between the affected and unaffected sides in BPPV patients before repositioning, indicating that BPPV could be the result of metabolic dysfunctions of bilateral otolith organs, which were in agreement with the findings of previous studies (23, 24). In 2015, Kim et al. (25) investigated o-VEMP and c-VEMP in 112 BPPV patients and 50 healthy controls to explore the functions of utriculus and saccule in the acute phase and resolved phase, demonstrating that the percentages of patients with abnormal cVEMP and oVEMP did not significantly differ in BPPV patients (25). Therefore, we speculated that the occurrence of BPPV was associated with the diseases of bilateral utricular maculae (26–29). The pathogenesis of otoconia dislodgment still remains unclear. Several studies have suggested that degenerative diseases could lead to the decrease of a gelatinous layer of the otolithic membrane, which in turn induces the spontaneous dislodgment of the otoconia from the utricular or saccular maculae (30, 31). In addition, the anatomical study, as well as quantitative and qualitative investigations performed by Richard et al. in 5 samples of the temporal bone (TB) from BPPV patients revealed the following: (1) all the 5TBs showed ~50% loss of ganglion cells in the superior vestibular division; (2) three TBs showed 50% loss of neurons in the inferior vestibular division, and the other two TBs with abnormal saccular ganglion cells showed 30% loss of neurons in the inferior vestibular division. Therefore, the investigators speculated that the occurrence of BPPV could be associated with the loss or reduction of bilateral otolith organs in semicircular canal (27), which could also explain why the recurrence of unilateral BPPV can affect the contralateral or even bilateral ears of patients (6).

### Dynamic otolith organ functions changes in BPPV patients

Our results demonstrated that the deviations of SVH90° and SVV of DUC at 180°/s-0 were significantly lower immediately after repositioning than before repositioning. According to the current understanding, vertigo induced by head movement in BPPV could be explained by the migration of calcium carbonate granules induced by the fractionation of otoconia in utricular maculae (32). Therefore, the rationale for canalith repositioning is promoting the otoconia to restore the positions in the utriculus through a series of procedures that change the positions of the head and body (19, 33). Consistent with the findings of this study, Maristela Mian Ferreira investigated the SVV deviation degree in 20 BPPV patients before and after repositioning by the “bucket test” in 2017 and found that the SVV deviation was significantly lower immediately after repositioning than before repositioning (34).

Our results demonstrated that although the VAS scores of vertigo continuously decreased after repositioning in BPPV patients, the deviation angles of SVH90° and SVV of DUC at 180°/s-0 gradually increased, coming close to the levels before repositioning. In addition, the SVH45° deviation degree significantly increased 1 month after repositioning than before repositioning. Our findings were in agreement with that of Kim et al. (25) published in 2015. In their study, they re-examined the VEMP 2 months after repositioning in 59 out of the 102 BPPV patients who were initially included in the study. Their findings showed that the VEMP of the affected and unaffected ears did not significantly differ at 2 months after repositioning compared with the levels before repositioning, which indicated that in contrast to other peripheral vestibular diseases (such as vestibular neuritis) where the damaged VEMP could be restored, otolith organ dysfunction could be persistent in BPPV patients after repositioning. This could be because with the process of aging, the decrease in a gelatinous layer of the otolithic membrane could more easily induce the spontaneous dislodgment of the otoconia from bilateral saccular maculae (26). However, although the dislodged otoconia debris could influence the deviation degree of SVV/SVH, i.e., the functions of otolith organs, it cannot induce the BPPV-related symptoms. Consistent with these findings, it is widely acknowledged that reduction in bone mass, osteoporosis, and vitamin D deficiency could induce the occurrence of BPPV through the disorders related to calcium metabolism in vestibular organs (35). Therefore, we speculated that with the increase in age and emergence of disorders related to calcium metabolism, the occurrence of vestibular degeneration could induce the dysfunction in bilateral otolith organs, which is also observed in patients with unilateral BPPV. The accumulation of debris dislodged from utricular maculae could induce the BPPV-related symptoms to a certain degree.

### Limitations

First, this study mainly focused on the dynamic changes in the function of otolith organs in BPPV patients before and after repositioning, without paying attention to the function of otolith organs and residual vertigo, which we plan to further investigate in our future studies. Secondly, dynamic and static SVV/SVH could be used for indirect assessment of the function of otolith organs, where procedures are simple and not influenced by the ages and muscle strength of patients and are therefore worthy of promotion in clinical practices. However, only very few studies investigated SVV/SVH by VR auxiliary technology in clinical practices, and the reference ranges need to be further standardized in more studies. Finally, the sample size in the present study was relatively small, which could lead to certain biases in statistical analysis. In our future clinical studies, we plan to include more patients and use multiple methods to assess the functions of otolith organs, thus further improving the findings of the present study.

## Conclusion

Before repositioning, the otolithic organ function of BPPV patients was obviously impaired, with no significant difference between the healthy and affected ear. After repositioning, there was a transient recovery of otolithic organ dysfunction followed by a sustained decline to similar levels to before repositioning.

## Data availability statement

The raw data supporting the conclusions of this article will be made available by the authors, without undue reservation.

## Ethics statement

The studies involving human participants were reviewed and approved by the Zhou Kou Central Hospital Ethical permission committee. The patients/participants provided their written informed consent to participate in this study.

## Author contributions

JS, CZ, and QY contributed to the study conception and design. CZ and JS contributed to the analysis and interpretation of data. CZ and QY wrote the first draft of the manuscript. JS made critical revision for important intellectual content. All authors contributed to the material preparation, data collection, read, and approved the final manuscript.

## Conflict of interest

The authors declare that the research was conducted in the absence of any commercial or financial relationships that could be construed as a potential conflict of interest.

## Publisher's note

All claims expressed in this article are solely those of the authors and do not necessarily represent those of their affiliated organizations, or those of the publisher, the editors and the reviewers. Any product that may be evaluated in this article, or claim that may be made by its manufacturer, is not guaranteed or endorsed by the publisher.
